# Supplementary figures and images for: A low caffeine dose improves maximal strength, but not relative muscular endurance in either heavier-or lighter-loads, or perceptions of effort or discomfort at task failure in females
Source: PeerJ. 2020 May 14;8:e9144. doi: 10.7717/peerj.9144 (PMC7231502; doi:10.7717/peerj.9144)

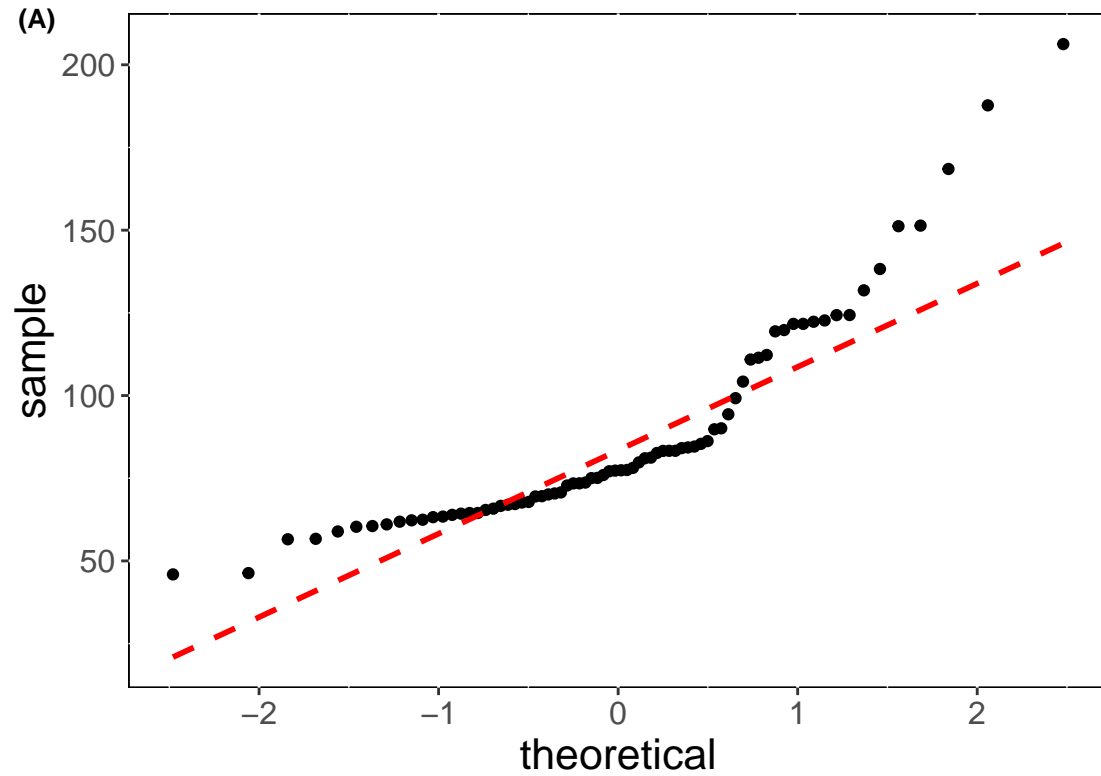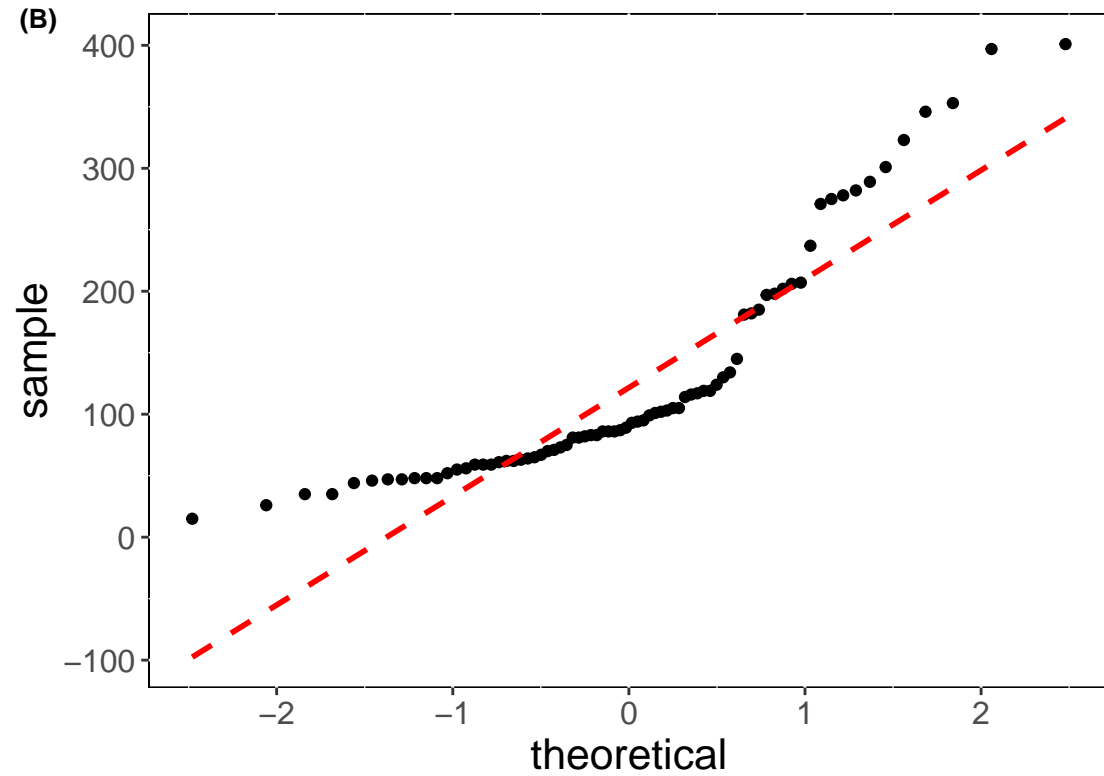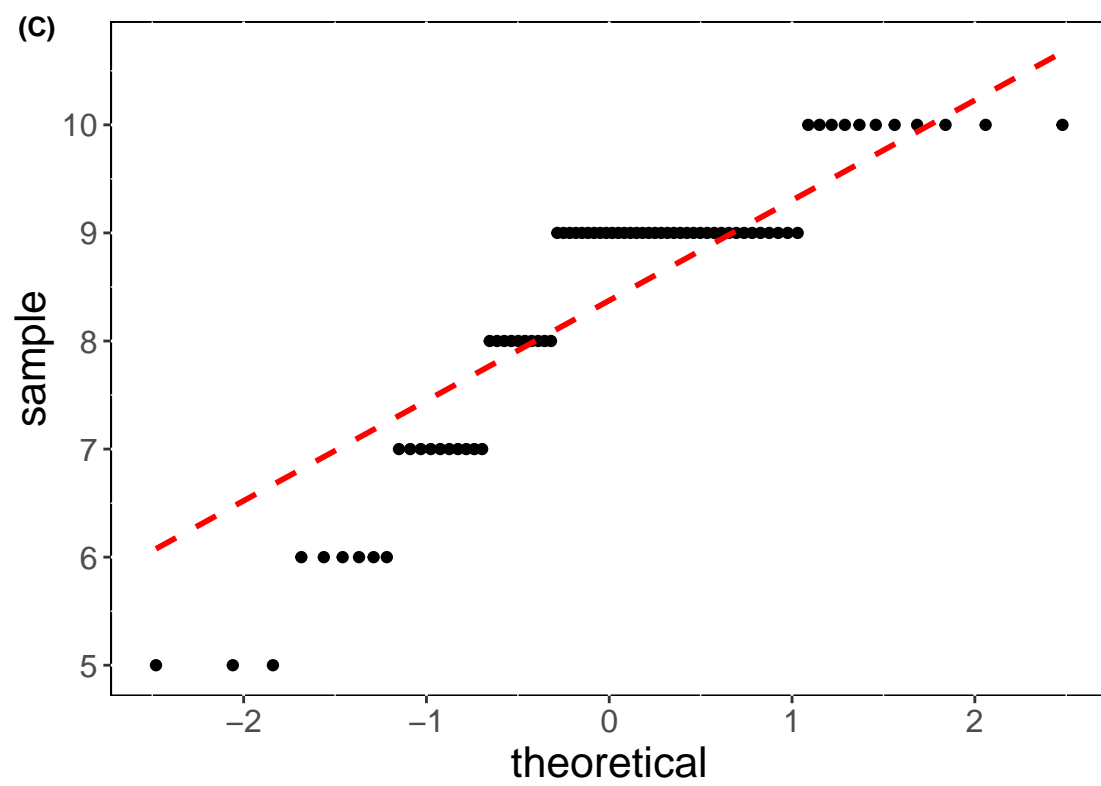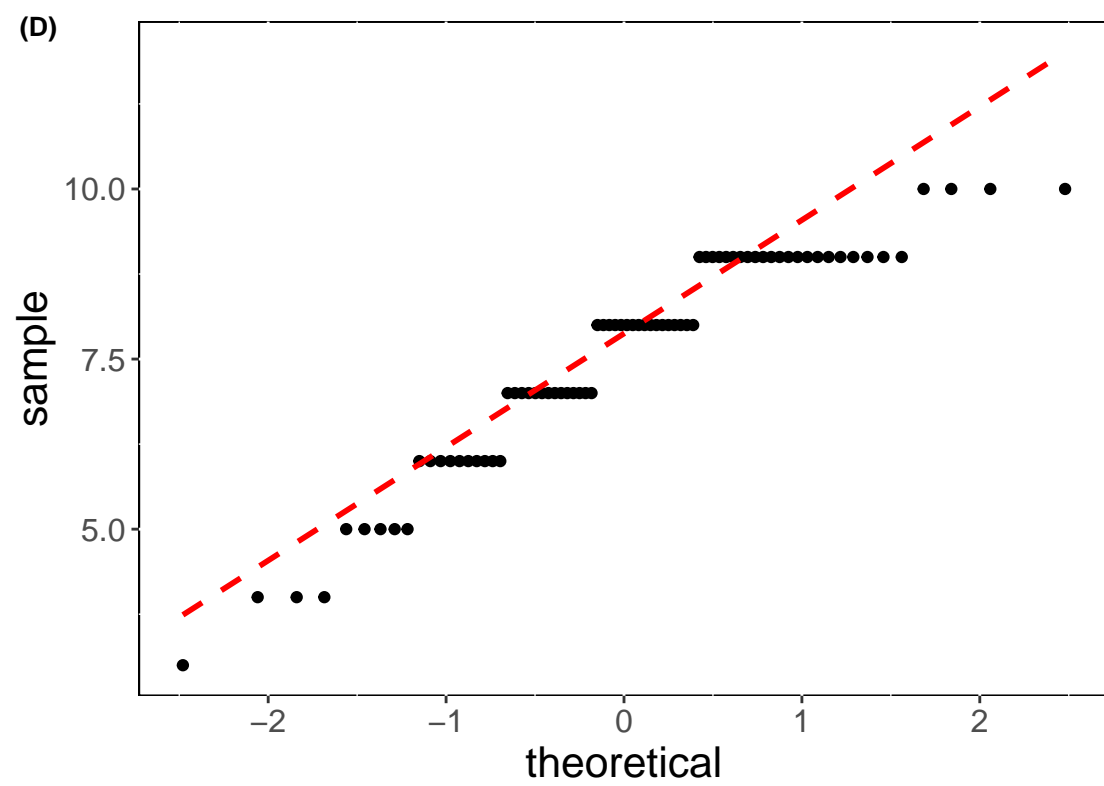

Supplement: Supplemental Information 3 [file peerj-08-9144-s003.pdf]
